# All Polymer Dielectric Films for Achieving High Energy Density Film Capacitors by Blending Poly(Vinylidene Fluoride-Trifluoroethylene-Chlorofluoroethylene) with Aromatic Polythiourea

**DOI:** 10.1186/s11671-020-3270-x

**Published:** 2020-02-06

**Authors:** Chengwei Li, Liuwei Shi, Wenyao Yang, Yujiu Zhou, Xiali Li, Chengguang Zhang, Yajie Yang

**Affiliations:** 1 State Key Laboratory of Electronic Thin Films and Integrated Devices, School of Optoelectronic Science and Engineering, University of Electronic Science and Technology of China, Chengdu, 610054 People’s Republic of China; 2 Chongqing Engineering Research Center of New Energy Storage Devices and Applications, Chongqing University of Arts and Sciences, Chongqing, 402160 People’s Republic of China

**Keywords:** Aromatic polythiourea, Composite films, Energy storage density, Molecular weight, Poly(vinylidene fluoride-trifluoroethylene-chlorofluoroethylene)

## Abstract

Construct dielectric films with high energy density and efficiency are the key factor to fabricate high-performance dielectric film capacitors. In this paper, an all organic composite film was constructed based on high dielectric polymer and linear dielectric polymer. After the optimized polycondensation reaction of a linear dielectric polymer aromatic polythiourea (ArPTU), the proper molecular weight ArPTU was obtained, which was introduced into poly(vinylidene fluoride-trifluoroethylene-chlorofluoroethylene) (PVDF-TrFE-CFE) terpolymer for a composite dielectrics. The results indicate that the addition of ArPTU molecules reduces the dielectric loss and improves the breakdown field strength of the PVDF-TrFE-CFE effectively. For the PVDF-TrFE-CFE/ArPTU (90/10) composite film, the maximum energy density about 22.06 J/cm^3^ at 407.57 MV/m was achieved, and high discharge efficiency about 72% was presented. This composite material can be casted on flexible substrate easily, and PVDF-TrFE-CFE/ArPTU organic composite films having high energy density, high breakdown field strength, low dielectric loss, and higher discharge efficiency are obtained. This is an unreported exploration about high energy density organic dielectric films based on PVDF-TrFE-CFE matrix and linear polymer dielectrics, and the findings of this research can provide a simple and scalable method for producing flexible high energy density materials for energy storage devices.

## Introduction

Dielectric film capacitors with high energy density, low dielectric loss, and high efficiency are required for compact and reliable power systems [1–7]. Among the available electrical energy storage technologies, dielectric film capacitors have the highest power density because of their ultra-fast charge and discharge capability [8, 9]. Energy storage dielectric materials play a vital role in dielectric film capacitors, the performance of dielectric films decides most performance of capacitors and constructing high energy density, and low dielectric loss dielectric films are attracting most attentions in related research. However, current dielectric materials exist in the dilemmas with having both high energy density and efficiency. Generally, polymers have high breakdown field strength but low dielectric constant [10, 11]. The energy density of biaxially oriented polypropylene (BOPP), which is widely used linear dielectric polymer today, is only 1.2 J/cm^3^, which is far from the needs of practical application. It is well known that ceramic materials have high dielectric constant, but the breakdown field strength is very low and the preparation process is complicated. It is already common to fill high dielectric constant inorganic nanomaterials into organic polymers for high energy density dielectrics. However, in many cases, the recombination of the two materials results in aggregation and interfacial adhesion due to the difference in compatibility between the two ingredients, resulting in high dielectric loss. To this end, new dielectric materials need to be sought and designed to further increase the energy density of the films and related devices.

Compared with inorganic materials, polymers are attractive materials that can be used as dielectrics [12–14] because of their simple processing technology and light density, resulting in lightweight and flexible films. As polymer dielectrics, poly(vinylidene fluoride) (PVDF) and its copolymers have been extensively studied for capacitor applications due to their high breakdown field and dielectric constant [15–19]. The high dipole moment of the C–F bond produces a PVDF-based polymer with higher dielectric constant. Unfortunately, the high remanent polarization and large hysteresis loss of PVDF and its copolymers limit their application on dielectric materials in capacitors. One way to solve this problem is to design a relaxed ferroelectric polymer with reduced hysteresis by incorporating structural defects into the PVDF matrix. For example, chlorofluoroethylene (CFE) is introduced into poly(vinylidene fluoride-trifluoroethylene) (PVDF-TrFE) to form poly(vinylidene fluoride-trifluoroethylene-chlorofluoroethylene) (PVDF-TrFE-CFE), and a narrow hysteresis loop and high dielectric constant are observed [20, 21]. However, PVDF terpolymers exhibit high dielectric loss under high electric field [22].

In recent years, linear dielectric polymers with polar groups have been utilized as high-performance polymer dielectrics due to the high breakdown field strength and discharge efficiency. More importantly, abundant linear dielectric polymers with different polar groups can be designed according to the first principle calculation for different applications [23]. Among these polymers, aromatic polythiourea (ArPTU) has been reported as a new linear dielectric polymers with high breakdown field strength (1.0 GV/m) and high charge and discharge efficiency (90% at 1.1 GV/m) [24, 25]. The aromatic polymer films still exhibit a linear dielectric response under high electric fields. Unlike other non-polar polymers, the random dipole and amorphous glass phase structure of polar groups in ArPTU can act as a trap, greatly increasing the scattering of carriers, thus greatly reducing conduction loss at high electric field. However, the ArPTU is brittle due to the rigid aromatic groups, making it unsuitable for large-area film preparation for dielectric film capacitor applications, especially the devices based on roll-to-roll processing. As for film preparation method, some new methods such as 3D printing appear for possible dielectric layer preparation [26, 27]. However, it needs further improvement before it can be applied to the film manufacturing process especially for large-area composite dielectrics.

In this paper, in order to solve these problems, a PVDF-TrFE-CFE/ArPTU all-organic dielectric material has been studied to achieve both high energy density and efficiency. Prior to the compounding process, the influence of molecular weight on performance of ArPTU was investigated in detail to meet the good synergistic effect between two polymers, and this would supply more valuable instruction to build high-performance and all-organic dielectrics based on linear dielectric materials. Then, by blending a small quantity of ArPTU into the PVDF-TrFE-CFE matrix, a simple solution casting method was utilized to prepare large-area composite films and composite dielectric films with high energy density and efficiency were achieved. In particular, this composite polymer is easy to process, lighter in weight, and lower in cost [28–30], which shows promising future as high-performance dielectric capacitor and energy storage applications.

## Materials and Methods

### Materials

PVDF-TrFE-CFE 63.2/29.7/7.1 (mol%) was purchased from Piezotech (France). 4,4′-Diphenylmethanediamine (MDA) was purchased from Aladdin (Shanghai, China), and *p*-phenylene diisothiocyanate (PDTC) was purchased from Acros (Belgium). *N*-Methylpyrrolidone (NMP) was supplied by Chengdu Kelong Chemical Company.

### Polythiourea Synthesis and Film Preparation

The ArPTU was synthesized by the polycondensation reaction. 1.922 g (0.01 mol) of PDTC and 1.982 g (0.01 mol) of MDA were added to a three-necked round bottom flask previously charged with 40 ml of NMP solvent under a N_2_ atmosphere. After reacting at room temperature for 6 h, it was washed with methanol for 3–5 times, and then dried in a vacuum oven at 60 °C for 12 h to obtain the polythiourea. By controlling the ratio of the two monomers of the synthetic polythiourea, three different molecular weight polythioureas of A, B, and C are obtained.

The PVDF-TrFE-CFE/ArPTU composite films with different proportions were prepared by a solution casting method. First, the pre-calculated mass of ArPTU and PVDF-TrFE-CFE was separately dissolved in NMP solvent to form the corresponding solution and stirred at room temperature for 4 h. Then, solutions of different mass ratios were separately mixed from the solution prepared in the previous step, and N_2_ was charged to avoid bubbles generated during the mixing, and stirred at room temperature for 6 h. The uniform thickness film was formed by solution casting method on the clean quartz glass plate, and the composite films were obtained by drying in a vacuum at 60 °C for 12 h.

### Electrical Performance Test

The unipolar polarization-electric field hysteresis loops of the dielectric polymer films were acquired using Precision Multiferroic (Radiant) equipped with 4000 V amplifier at room temperature and a frequency of 10 Hz. The efficiency of the charge-discharge cycle as a function of the applied field was given by the ratio of the discharged energy to the stored electric energy. Dielectric constant and loss of the dielectric polymer films were measured in a 100-Hz to 1-MHz range at room temperature with Impedance Analyzer (Agilent 4294A). The breakdown field strength of the dielectric polymer films was measured by AC and DC withstand voltage insulation resistance tester (TH9201) at room temperature. The breakdown strength of the composite films was determined by Weibull distribution statistics.

### Characterization of the Materials

Scanning electron microscope (SEM, Hitachi S-4800) was used to observe the surface morphology of the dielectric polymer films. The Fourier-transform infrared spectroscopy (FTIR) curves of the dielectric polymer films were observed by FTIR spectrometer (8400S, Shimadzu) in the range of 400 to 4000 cm^−1^. The X-ray diffraction (XRD) patterns of the dielectric polymer films were recorded by an X-ray powder diffractometer (X’Pert Pro, Panalytical) using Cu Kα radiation.

## Results and Discussions

### Dielectric Properties of Different Molecular Weight ArPTU Films

**Fig. 1:**
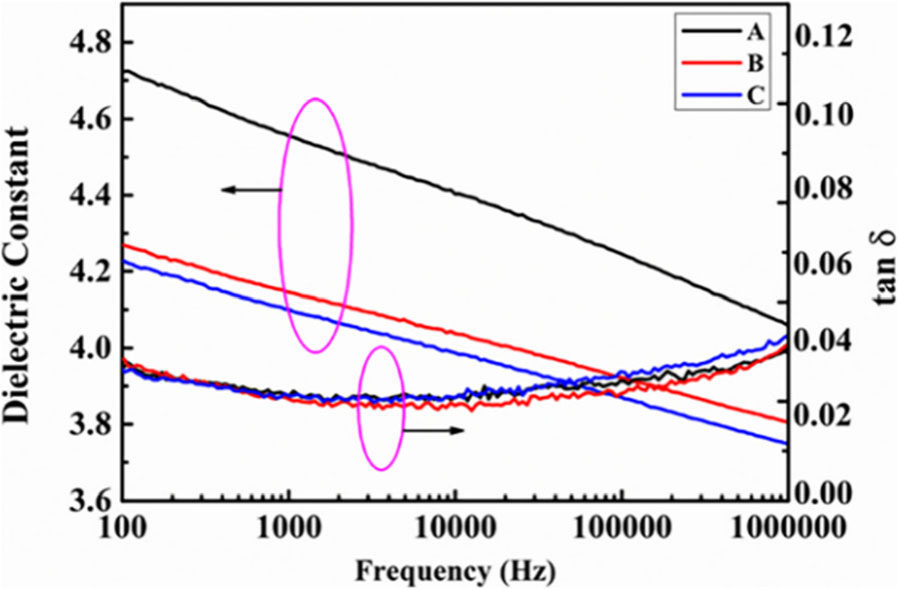
The dielectric constant and dielectric loss of different molecular weight ArPTU films (A, B, and C are polythioureas synthesized by the molar ratio PDTC/MDA (1/1), PDTC/MDA (0.95/1), and PDTC/MDA (1.05/1))

Molecular weight shows distinct influence on physical performance of ArPTU, especially the dielectric performance and processability. By controlling the polycondensation reaction conditions, especially the ratio of the two monomers, ArPTU with different molecular weight were synthesized, as shown in Table 1 (A, B, and C are polythioureas synthesized by the molar ratio PDTC/MDA (1/1), PDTC/MDA (0.95/1), and PDTC/MDA (1.05/1)). By tuning the molar ratio of the two monomers MDA and PDTC, the weight average molecular weight and number average molecular weight of three ArPTU decreased successively in the order of A > B > C. Figure 1 shows the dielectric constant and dielectric loss of different molecular weight ArPTU films as a function of frequency. It can be seen that the dielectric constant of different molecular weight ArPTU films decrease with increasing frequency. This is because the ArPTU molecule has a polar group-thiourea group, and the dipoles turning polarization in molecules contribute a lot to the dielectric constant. With the test frequency increasing, the contribution of dipole steering polarization decreases [31]. Especially at high frequency, the speed of dipole steering cannot keep up with the change of the electric field, resulting in the dielectric constant decreases with increasing test frequency.

**Table 1:** Molecular weights of three different polythioureas

Sample	Weight-average molecular weight	Number-average molecular weight	*Z*-average molecular weight
A	68,476	31,582	111,752
B	30,102	13,612	48,002
C	21,321	8564	34,776

At the test frequency of 1000 Hz, the dielectric constant of different molecular weight ArPTU films decreases in the order of A (4.55) > B (4.15) > C (4.10), which is consistent with the order of molecular weight of the three ArPTU. The reason for this phenomenon may result from the coordinated orientation of the ArPTU grain boundary layer dipole in large molecular weight polymers [32, 33]. In this molecular structure, the molecular segment of the ArPTU grain boundary layer not only maintains the alignment characteristics of the crystalline region molecules, but also not limited by the lattice network. Therefore, in the ArPTU films, the higher the volume fraction of the grain boundary layer, the higher the dielectric constant. The high molecular weight ArPTU film containing more long-chain molecules and the grain boundary layer will also occupy more volume, resulting higher dielectric constant.

As shown in (Fig. 1), the dielectric loss of different molecular weight ArPTU films decreases firstly and then increases with the increasing of test frequency. In the 100–10,000 Hz domain, the DC ion conductance decreases with increasing test frequency, resulting in a decrease in dielectric loss. When test frequency is higher than 10,000 Hz, the dipole relaxation causes the dielectric loss to increase with the increasing of the test frequency [34]. Obviously, the dielectric loss curves of the three samples are not much different, but there is only a small difference in the high-frequency region. In other words, the molecular weight of the ArPTU has little effect on dielectric loss of ArPTU films.

**Fig. 2:**
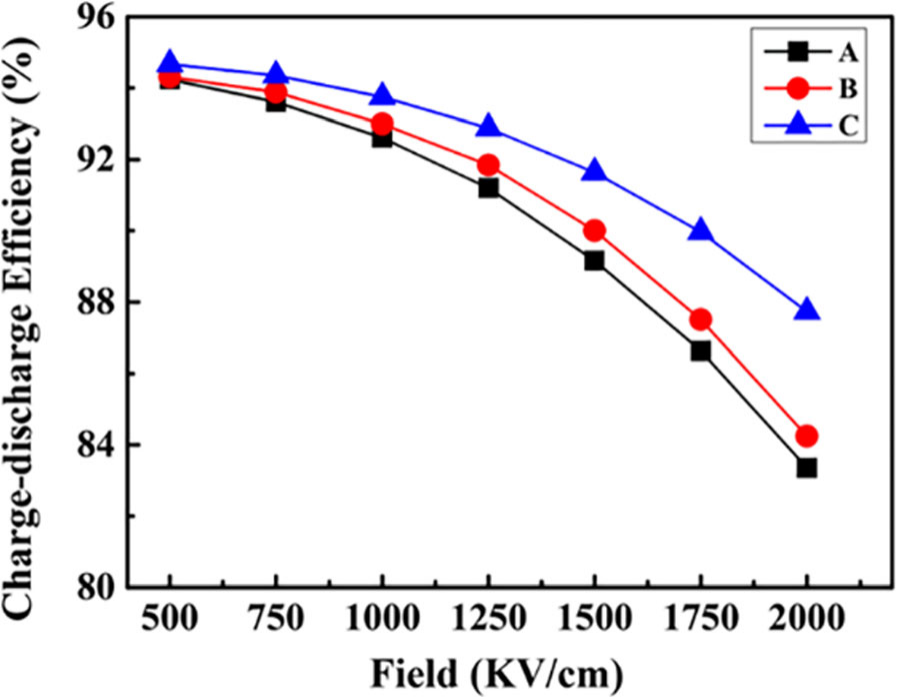
The charge-discharge efficiency of different molecular weight ArPTU films (A, B, and C are polythioureas synthesized by the molar ratio PDTC/MDA (1/1), PDTC/MDA (0.95 /1), and PDTC/MDA (1.05/1))

The charge-discharge efficiency of different molecular weight ArPTU films can be calculated by measuring the unipolar polarization-electric field hysteresis loops, as shown in (Fig. 2). The charge-discharge efficiency decreases with the increase of the applied electric field. Compared with the high molecular weight ArPTU film, the charge-discharge efficiency of the low molecular weight ArPTU film decreases at a slower rate. Under the electric field of 2000 KV/cm, the charge-discharge efficiency of different molecular weight ArPTU films increased in the order of A (83.35%) < B (84.24%) < C (87.73%), the low molecular weight ArPTU film has higher charge-discharge efficiency. This is because the high molecular weight ArPTU film contains more long-chain molecules forming larger-sized ferroelectric domains when crystallizes, and larger-size ferroelectric domains result in higher residual polarization and lower charge-discharge efficiency accordingly.

**Fig. 3:**
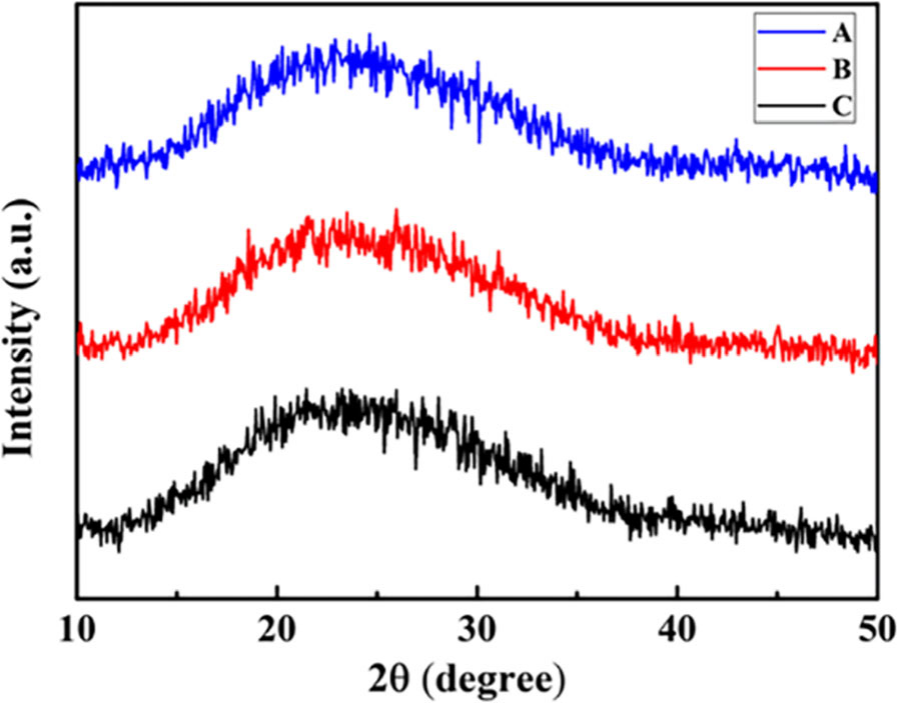
The XRD curves of different molecular weight ArPTU films (A, B, and C are polythioureas synthesized by the molar ratio PDTC/MDA (1/1), PDTC/MDA (0.95/1), and PDTC/MDA (1.05/1))

Figure 3 is the XRD curves of different molecular weight ArPTU films. The ArPTU films with different molecular weights have relatively wide X-ray diffraction peaks at 2θ ≅ 22°, and the intensity of the peaks decreases with the increase of molecular weight. This is because ArPTU has an amorphous structure, and the higher molecular weight ArPTU film contains more long-chain molecules, resulting in larger amorphous region. Accordingly, the crystallinity in polymer films lowered, which results in the weakening of the diffraction peak [35, 36].

### Characterization of PVDF-TrFE-CFE/ArPTU Composite Films

**Fig. 4:**
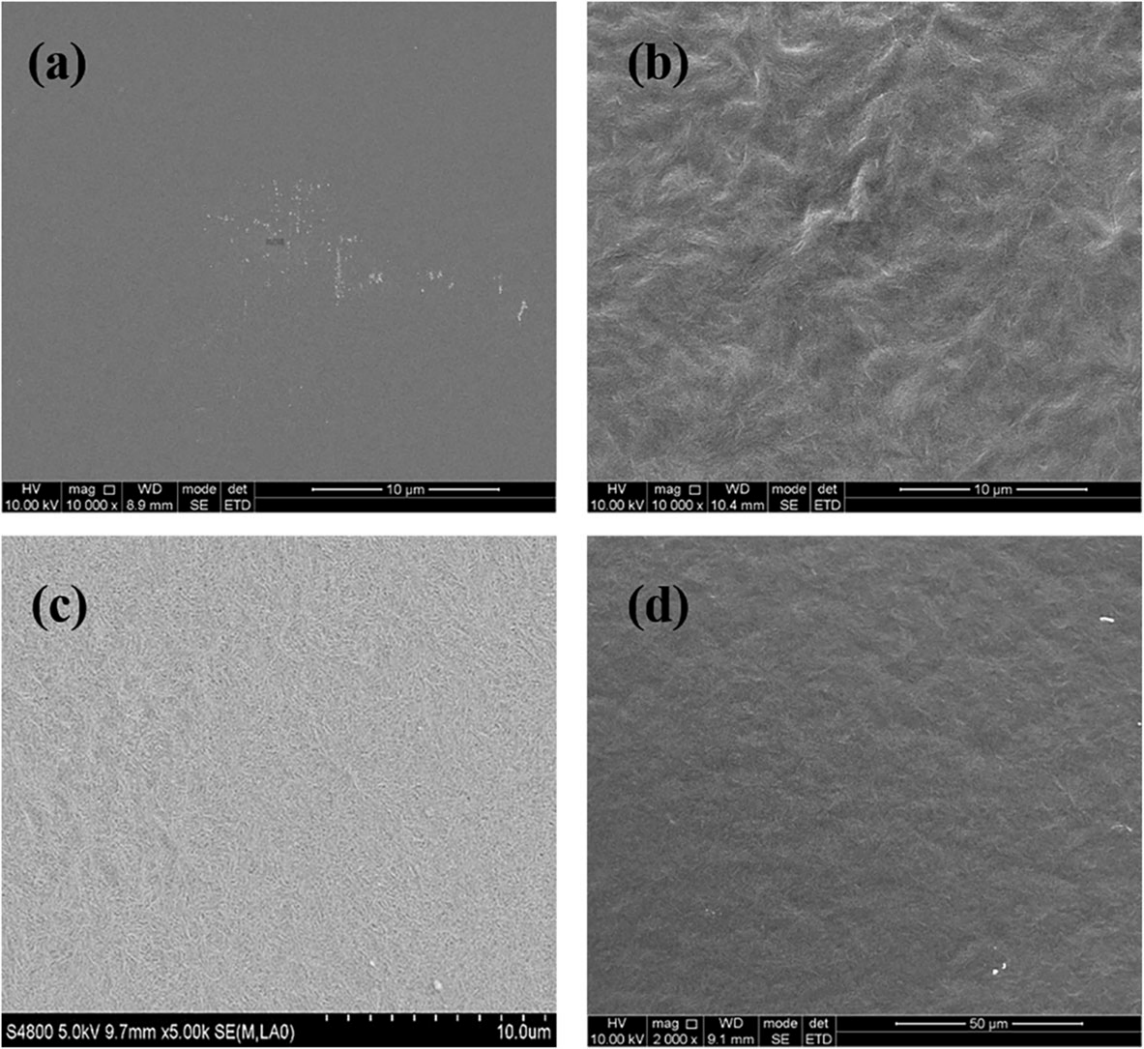
SEM image of different films. **a** ArPTU. **b** PVDF-TrFE-CFE/ArPTU (90/10). **c** PVDF-TrFE-CFE. **d** PVDF-TrFE-CFE/ArPTU (95/5)

Figure 4 shows the surface morphology of ArPTU, PVDF-TrFE-CFE, and PVDF-TrFE-CFE/ArPTU (90/10) characterized by scanning electron microscopy (SEM). It can be observed that the surface of the PVDF-TrFE-CFE film represents a dendritic structure, indicating its high crystallinity, which consists of the XRD results. The ArPTU film shows very smooth film surface, and some small particles appear on the surface of the PVDF-TrFE-CFE/ArPTU (90/10) composite film. Obviously, the domains of PVDF-TrFE-CFE have been reduced by blending ArPTU, which also consists of the XRD data.

**Fig. 5:**
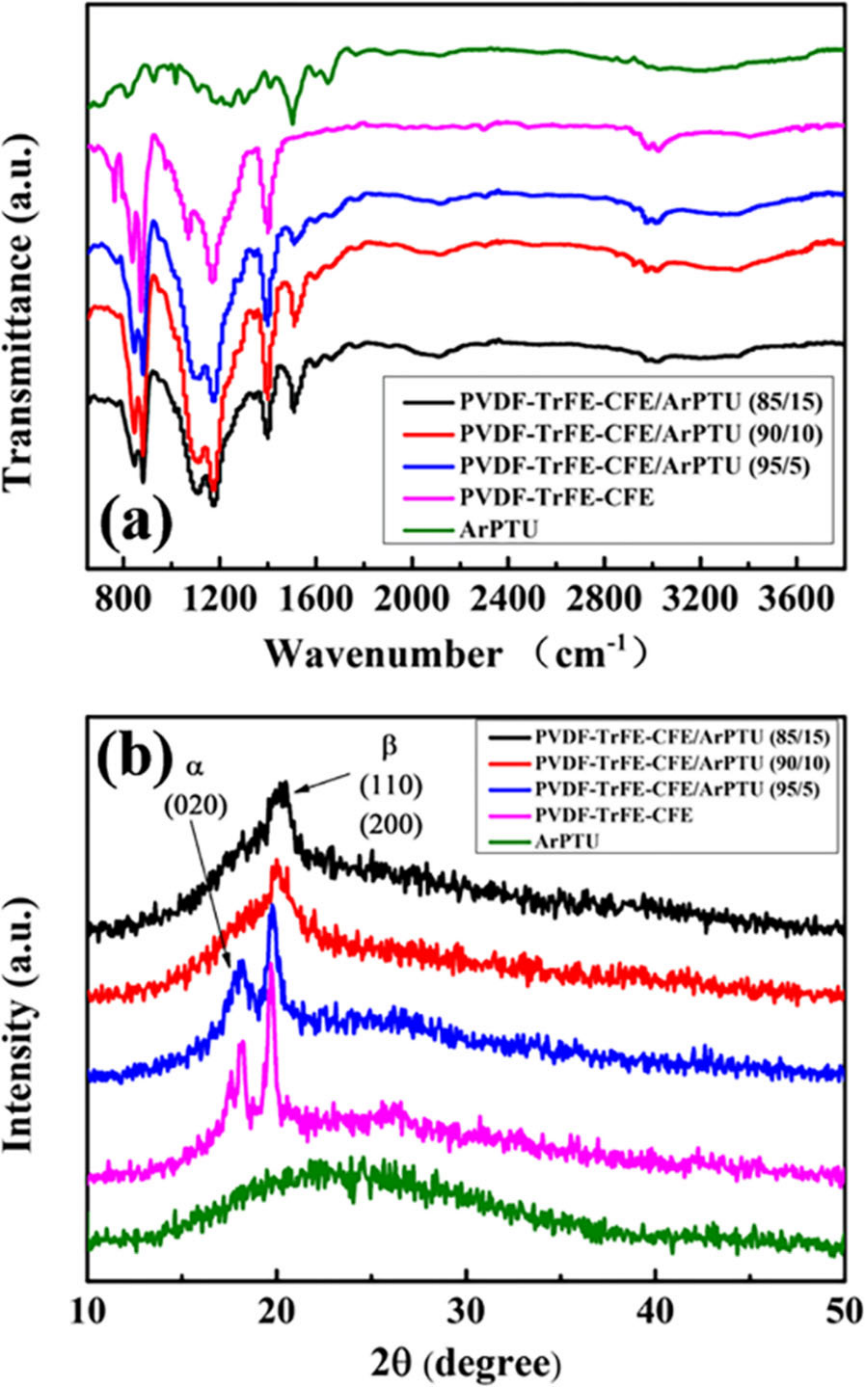
**a** The FTIR curves of PVDF-TrFE-CFE/ArPTU composite films with different composite ratios. **b** The XRD curves of PVDF-TrFE-CFE/ArPTU composite films with different composite ratios

The FTIR curves of PVDF-TrFE-CFE/ArPTU composite films with different ArPTU mass ratios are shown in (Fig. 5a). The FTIR curves show that the composite films with different ratios have obvious absorption peaks at 1230 cm^−1^, resulting from the -HN-CS-NH- group in polythiourea, which proves the existence of ArPTU in the composite films. The XRD curves of PVDF-TrFE-CFE/ArPTU composite films with different compounding ratios are shown in (Fig. 5b). It can be seen that the PVDF-TrFE-CFE film and PVDF-TrFE-CFE/ArPTU composite films have obvious characteristic peaks at 2θ ≅ 19.72°, and this peak is a characteristic diffraction peak of the β-phase (110) and (200) crystal planes. The intensity of the diffraction peak decreases with the increase of ArPTU content, which means that the crystallinity of the composite film decreases with the ArPTU content increasing. In addition, PVDF-TrFE-CFE film and PVDF-TrFE-CFE/ArPTU (95/5) film have a weaker diffraction peak at 2θ ≅ 17.56°, and this peak is the characteristic diffraction peak of the α-phase (020) crystal plane. When the mass fraction of ArPTU reaches more than 10%, the crystallization peak of the α-phase of PVDF-TrFE-CFE/ArPTU composite films weakened, indicating that composite films transform to an amorphous state slowly with the increasing of ArPTU component.

### Dielectric Properties of PVDF-TrFE-CFE/ArPTU Composite Films

**Fig. 6:**
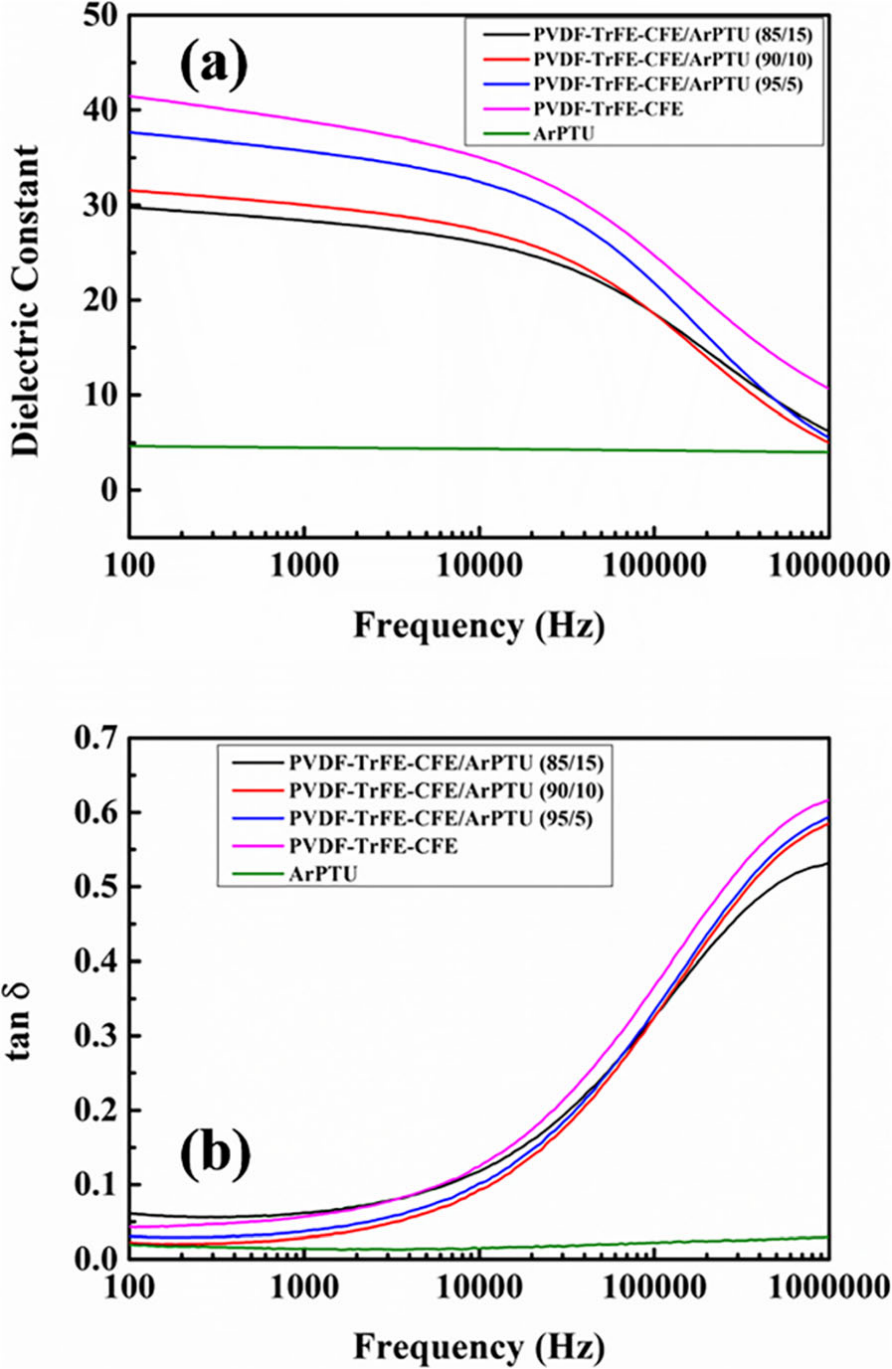
**a** Dielectric constant of ArPTU, PVDF-TrFE-CFE, and PVDF-TrFE-CFE/ArPTU composite films. **b** Dielectric loss of ArPTU, PVDF-TrFE-CFE, and PVDF-TrFE-CFE/ArPTU composite films

Based on the results above, the ArPTU sample B, which has higher dielectric constant and discharge efficiency, was chosen to prepare composite dielectric films with PVDF-TrFE-CFE. Firstly, to study the influence of ArPTU on the dielectric property of PVDF-TrFE-CFE matrix, dielectric frequency spectra in a 100-Hz to 1-MHz range were characterized at room temperature. As presented in (Fig. 6a), it can be seen that the dielectric constant of the composite films gradually decreases as the content of ArPTU increases. The composite films have dielectric constants of 35.72, 30.02, and 28.37 at 95/5, 90/10, and 85/15 ratios in 1000 Hz, respectively. The reduced dielectric constant of the composite films is due to the addition of low dielectric constant ArPTU. At the same time, with the amount of ArPTU addition increasing, the dielectric constant frequency dependence of the composite films decreases. This is because the thiourea units in the ArPTU interact with the PVDF-TrFE-CFE matrix, which limits the rotation of the dipoles in PVDF-TrFE-CFE [37].

Figure 6b shows the relationship between dielectric loss and frequency of the composite films with different ArPTU ratios. It can be seen that the dielectric loss of all composite films is lower than PVDF-TrFE-CFE film, indicating that the addition of ArPTU molecules can reduce the dielectric loss of the PVDF-TrFE-CFE effectively. This is attributed to the thiourea unit in polythiourea increasing the interplanar space, and the dipoles in the polymer chain have more space to rotate freely, which limits dipole relaxation effectively. Since the dielectric loss under high frequencies mainly comes from dipolar relaxation, the results again indicate that the thiourea groups in ArPTU may confine dipole relaxation [37, 38].

**Fig. 7:**
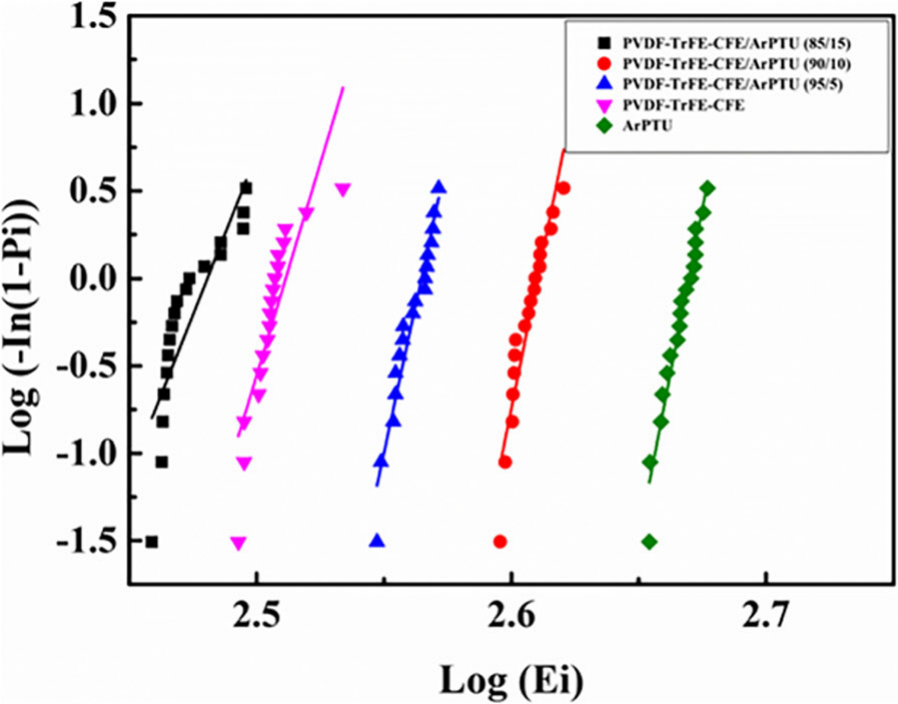
Weibull breakdown of ArPTU, PVDF-TrFE-CFE, and PVDF-TrFE-CFE/ArPTU composite films

The breakdown field strength of dielectric films is another important parameter for practical capacitor applications. The breakdown field strength of the composite films with different ArPTU ratios is characterized by Weibull distribution statistics, which are shown in (Fig. 7). For ArPTU, PVDF-TrFE-CFE, PVDF-TrFE-CFE/ArPTU (95/5), PVDF-TrFE-CFE/ArPTU (90/10), and PVDF-TrFE-CFE/ArPTU (85/15) films, the breakdown field strength calculated by Weibull distribution were 467.5 MV/m, 324.6 MV/m, 366.9 MV/m, 407.6 MV/m, and 302.4 MV/m, respectively. It reveals that compared with the PVDF-TrFE-CFE film, the breakdown field strength of the composite films is significantly improved by introduction of ArPTU, and the more ArPTU content contains, the higher breakdown field strength of composite film is obtained. The addition of ArPTU enhances electron-phonon scattering and electron-dipole scattering in the composite films, which results in the improved breakdown field significantly [38]. However, when the ArPTU content is increased to 15%, the breakdown field strength of composite reduces, which may be due to the delamination phenomenon of two polymers, resulting in more defects in composite and the reduction of breakdown field strength accordingly. Hence, the proper addition of ArPTU will improve the breakdown field strength of high dielectric PVDF-TrFE-CFE films effectively.

**Fig. 8:**
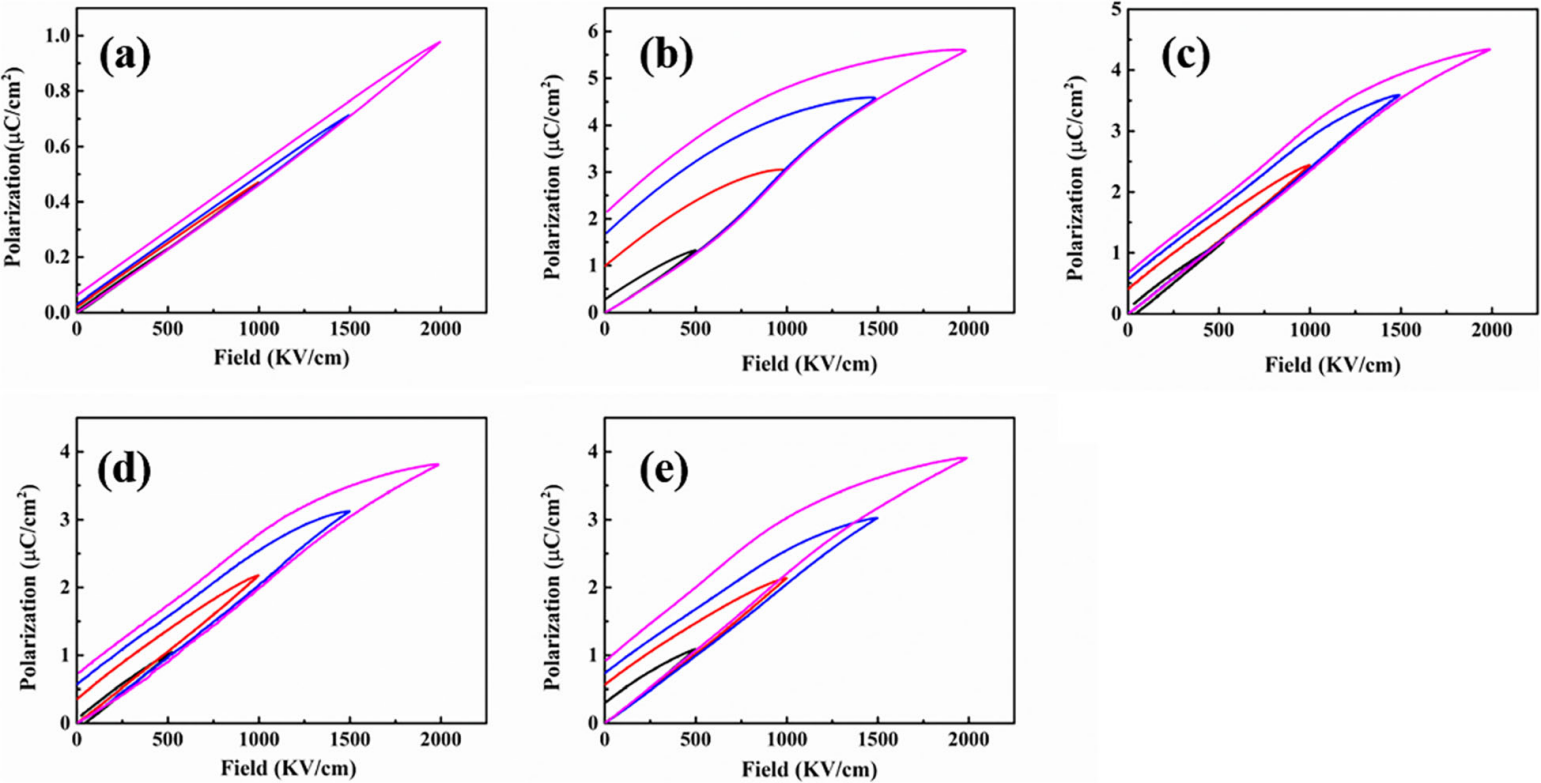
Unipolar polarization-electric field hysteresis loops. **a** ArPTU. **b** PVDF-TrFE-CFE. **c** PVDF-TrFE-CFE/ArPTU (95/5). **d** PVDF-TrFE-CFE/ArPTU (90/10). **e** PVDF-TrFE-CFE/ArPTU (85/15)

The unipolar polarization-electric field hysteresis loops of PVDF-TrFE-CFE/ArPTU composite films with different ArPTU ratios are shown in (Fig. 8). The maximum polarization of the composite films decreases with the increase of ArPTU content. The residual polarization of the composite films with three different ratios decreases relative to the PVDF-TrFE-CFE film, indicating that the addition of ArPTU molecules can effectively inhibit the early polarization saturation of PVDF-TrFE-CFE, resulting in higher charge-discharge efficiency.

**Fig. 9:**
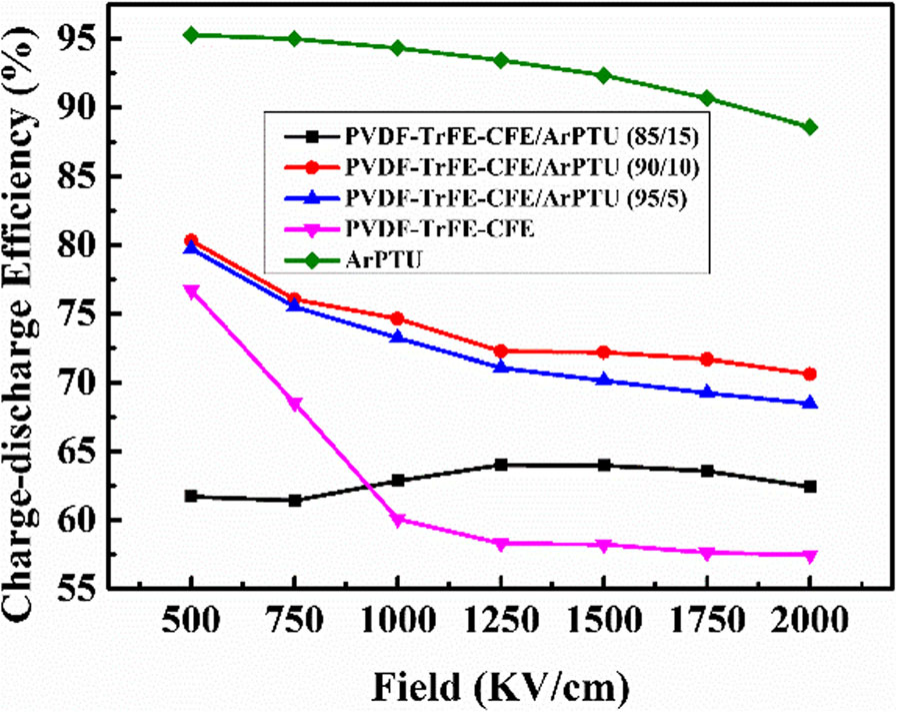
Charge-discharge efficiency of PVDF-TrFE-CFE/ArPTU composite films with different composite ratios

In practical applications, the charge-discharge efficiency is another important characteristic parameter of dielectric materials due to the loss of energy which always leads to heating and damages the performance and reliability of the capacitor. Figure 9 shows the charge-discharge efficiency of the PVDF-TrFE-CFE/ArPTU composite films with different ArPTU ratios. The applied field strength of the PVDF-TrFE-CFE film increased from 500 to 2000 KV/cm, and the charge-discharge efficiency decreased from 77 to 58%, mainly due to the ferroelectric hysteresis loss under high electric field. The charge-discharge efficiency of composite films with different ArPTU ratios is significantly higher than that of the PVDF-TrFE-CFE film. The PVDF-TrFE-CFE/ArPTU (90/10) film maintains 72% charge-discharge efficiency at an electric field of 2000 KV/cm. At 2000 KV/cm, the composite shows a high energy density with 5.31 J/cm^3^, which is much higher than BOPP films for practical use. The addition of ArPTU changes the molecular structure of PVDF-TrFE-CFE and inhibits PVDF-TrFE-CFE from reaching polarization saturation prematurely. It has been also found that the proper addition ratio of ArPTU show distinct influence on charge-discharge efficiency of composite films. The 85/15 ratio composite has a relatively low charge-discharge efficiency due to the high ArPTU content, which may result from the delamination phenomenon of two polymers.

**Fig. 10:**
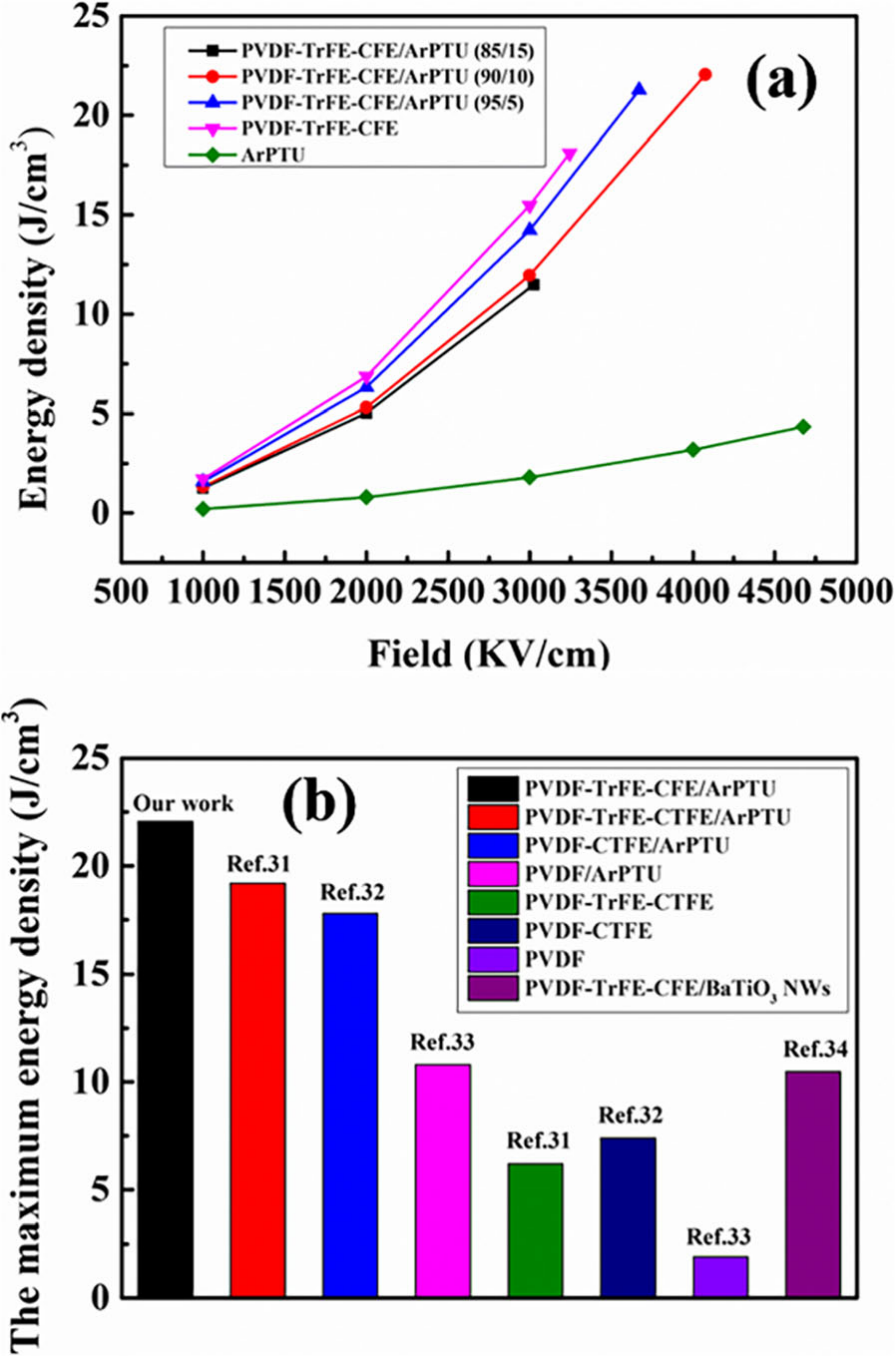
**a** The energy density of PVDF-TrFE-CFE/ArPTU composite films with different composite ratios. **b** Comparing of discharge energy density of our works with reported works [39, 40]

The energy density of PVDF-TrFE-CFE/ArPTU composite films with different composite ratios is shown in Fig. 10a. The improvement of the storage density of the composite films relative to the ArPTU film is consist of the result of the dielectric constant performance of the composite films. It can be seen that compared with the pure ArPTU film, the PVDF-TrFE-CFE/ArPTU composite films have higher energy density at the same electric field because of the enhanced dielectric constant. The maximum energy density of the PVDF-TrFE-CFE film in PVDF-TrFE-CFE/ArPTU (90/10) composite film has a storage density of 22.06 J/cm^3^ at 4076 KV/cm. Compared with PVDF-TrFE-CTFE/ArPTU composite films (19.2 J/cm^3^) [37], the film in our work shows higher energy storage density. Although the films in our work show slightly lower breakdown voltage, higher dielectric constant ensures great enhancement of energy storage density. So, a tradeoff of breakdown strength and dielectric constant should be considered when constructing high energy density composite films. Furthermore, considering the discharge energy density, our work also indicates high competitiveness with highest discharge energy density, which is shown in Fig. 10b. Compared to the organic-inorganic composite films, the organic composite films can improve energy storage density and efficiency of the film more efficiently and are feasible in practical applications for roll-to-toll device fabrication [41, 42]. In all, by controlling the molecular weight and addition ratio of ArPTU properly, high-performance organic dielectrics based on PVDF-TrFE-CFE/ArPTU with high energy density, high breakdown field strength, low dielectric loss, and higher charge-discharge efficiency can be constructed. This high-performance polymer film has been proven to be promising dielectric materials for high-power density film capacitor applications.

## Conclusion

ArPTU was introduced into PVDF-TrFE-CFE/ArPTU to prepare composite dielectric films through a solution casting method. Compared with PVDF-TrFE-CFE film, PVDF-TrFE-CFE/ArPTU composite films have higher breakdown field strength, higher charge and discharge efficiency, and lower dielectric loss. A higher breakdown field strength means an increase in the energy storage density. The PVDF-TrFE-CFE/ArPTU (90/10) composite film has a storage density of 22.06 J/cm^3^ at 407.57 MV/m. The improvement in dielectric properties of the composite films is related to changes in crystal structure. The excellent dielectric properties and simple preparation process of PVDF-TrFE-CFE/ArPTU composite films make it an important research breakthrough for future dielectric materials and a promising application prospect for energy storage devices.

## Data Availability

All datasets are presented in the main paper or in the additional supporting files.

## Abbreviations

AC: Alternating current
ArPTU: Aromatic polythiourea
BOPP: Biaxially oriented polypropylene
CFE: Chlorofluoroethylene
DC: Direct current
MDA: 4,4′-Diphenylmethanediamine
NMP: *N*-Methylpyrrolidone
PDTC: *p*-Phenylene diisothiocyanate
PVDF: Poly(vinylidene fluoride)
PVDF-TrFE: Poly(vinylidene fluoride-trifluoroethylene)
PVDF-TrFE-CFE: Poly(vinylidene fluoride-trifluoroethylene-chlorofluoroethylene)
PVDF-TrFE-CTFE: Poly(vinylidene fluoride-trifluoroethylene-chlorotrifluoroethylene)
